# Oral Microbiome-Mediated Carcinogenesis in Oral Cavity Cancer: A Narrative Review

**DOI:** 10.7759/cureus.83242

**Published:** 2025-04-30

**Authors:** Volkan Semiz, Rahmi A Aksoy, Zekiye Altun

**Affiliations:** 1 Radiation Oncology, İzmir City Hospital, Izmir, TUR; 2 Radiation Oncology, Izmir City Hospital, Izmir, TUR; 3 Basic Oncology, Dokuz Eylül University Institute of Oncology, Izmir, TUR

**Keywords:** carcinogenesis, dysbiosis, inflammation, microbiome, oral cavity cancer

## Abstract

The global impact of oral cavity cancer (OCC) is substantial, given its five-year survival rate of nearly 50%. Tobacco, alcohol, and betel nut consumption are primary risk factors, but OCC can also develop in individuals without these exposures. Recent studies increasingly indicate that the oral microbiome may contribute to the pathogenesis of OCC, especially in the context of poor oral hygiene and periodontal disease. The oral microbiome consists of hundreds of bacterial and fungal species, influenced by factors such as smoking, alcohol, diet, and medications. Smoking disrupts microbial balance and epithelial barriers, contributing to dysbiosis. Studies have linked periodontal pathogens like *Porphyromonas gingivalis*, *Fusobacterium nucleatum*, and *Treponema denticola* to OCC, while certain *Streptococcus* species may have protective effects. Microbial alterations are observed in both OCC and precancerous lesions, suggesting a role in early carcinogenesis. The oral microbiome may facilitate carcinogenesis through multiple interrelated mechanisms, including the generation of carcinogenic byproducts (e.g., nitrosamines, acetaldehyde, hydrogen sulfide), persistent inflammatory signaling, immune evasion, resistance to apoptosis, and the activation of epithelial-mesenchymal transition pathways. These factors contribute to DNA damage, genomic instability, and tumor progression. A deeper understanding of how the oral microbiome influences OCC may offer novel strategies for both prevention and clinical management. Future research should focus on microbiome-targeted interventions to reduce OCC risk and improve clinical outcomes.

## Introduction and background

**Figure 1:**
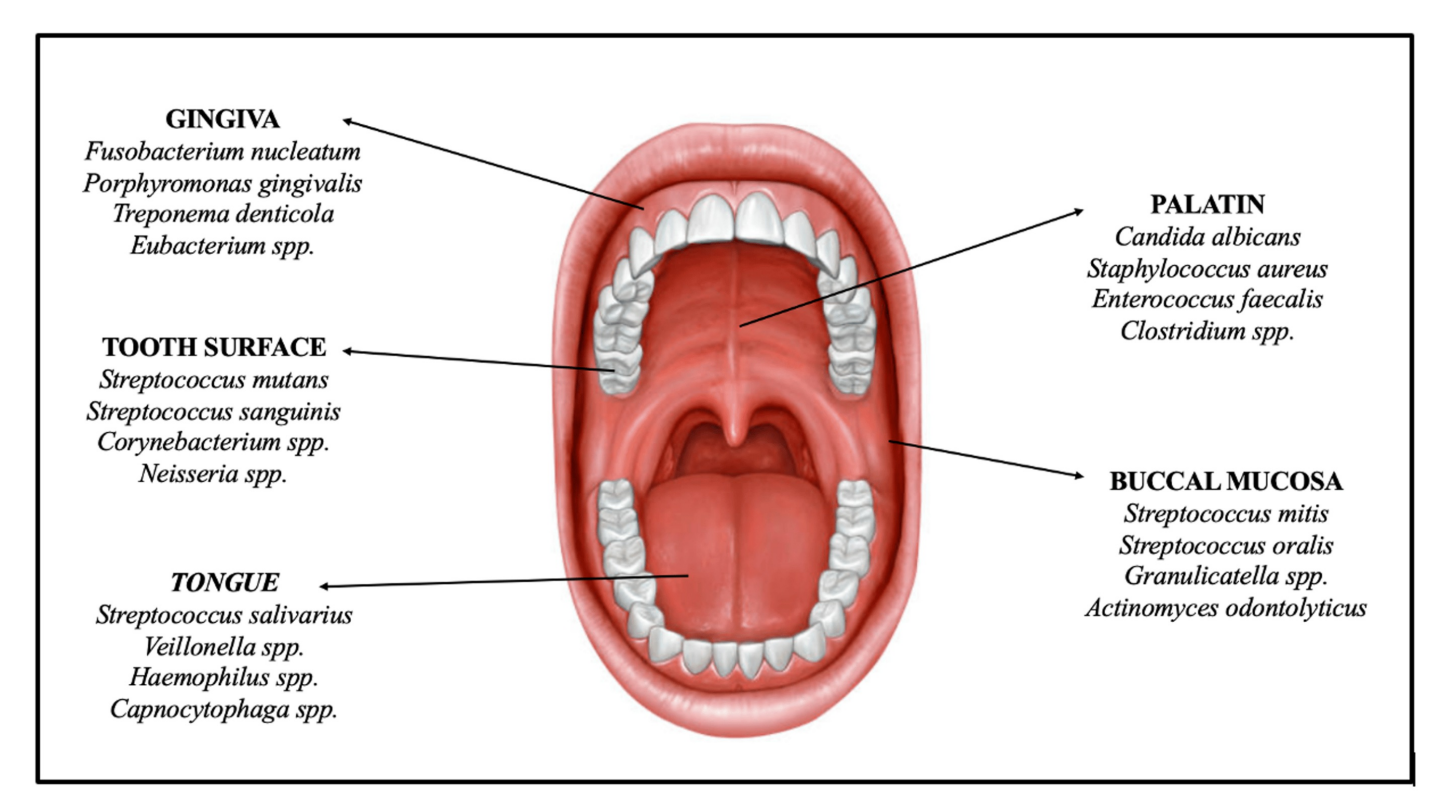
Anatomical distribution of the oral microbiome: Key colonization sites of bacteria and fungi. This image was created with BioRender (https://biorender.com/).

Oral cavity cancers (OCC) are recognized as the eighth most common cancer worldwide, but in South-Central Asia, they rank among the top three cancers. Despite advances in treatment, the five-year overall survival rate for OCC remains around 50% [1]. The most significant risk factors identified in OCC development are tobacco, alcohol, and betel nut use, with approximately 80% of patients being smokers [2,3]. The combined use of tobacco and alcohol significantly increases the risk of OCC development. Additionally, OCC can develop in some patients even without the use of tobacco or alcohol. Studies have shown that poor oral hygiene and tooth loss are associated with an increased risk of OCC [4]. This association indicates a potential role of the oral microbiome in the carcinogenic process of OCC. The oral microbiome consists of approximately 700 species of bacteria and 100 species of fungi. These microorganisms inhabit various areas of the oral cavity, including the teeth, gums, tongue, cheeks, hard and soft palates. Moreover, they extend to structures such as the throat, nasal cavity, sinuses, and larynx [5]. Figure 1 illustrates the anatomical distribution of the oral microbiome, highlighting the predominant colonization sites of bacterial and fungal species within the oral cavity.

In healthy individuals, the oral cavity microbiome does not exhibit the same level of microbial diversity as seen in the gastrointestinal system. However, it is well-established that several factors can influence the oral microbiome. The most significant factors include tobacco and alcohol use, dietary habits, obesity, various medications, and environmental influences [6]. Studies examining smoking-induced changes in the oral microbial environment have shown that it disrupts both the microbiome and the epithelial barrier [7,8]. Oral dysbiosis can be associated with many diseases, with the most well-known being periodontal diseases, halitosis, oral candidiasis, and dental problems [9]. The link between periodontal diseases and OCC has also been demonstrated in meta-analyses [10,11].

Recent advances have considerably deepened our insight into how the oral microbiome interacts with both oral and systemic health. A growing body of research indicates that dysbiosis within the oral microbial community may be implicated not only in localized conditions such as periodontal disease but also in the pathogenesis of malignancies like OCC. In this context, the present review aims to critically examine the potential biological pathways through which the oral microbiome may contribute to the development and progression of OCC.

## Review

Methods

This narrative review explores the association between the oral microbiome and OCC. A comprehensive literature search was conducted in the PubMed and Google Scholar databases using the following keywords: microbiome AND oral cancer; microbiome AND oral cavity cancer; microbiota AND oral cancer; microbiota AND oral cavity cancer. The search primarily focused on studies published in the last ten years, though older, relevant articles cited in the retrieved literature were also considered. Inclusion criteria required that all articles be published in English, and eligible study types included clinical trials, case-control studies, retrospective studies, prospective studies, and review articles. Articles published in languages other than English and editorials were excluded. After an initial screening, studies unrelated to the topic were excluded. In addition, the reference lists of the included articles were manually screened to identify additional relevant studies. Duplicate records were removed to ensure the inclusion of unique and relevant studies.

The relationship between the microbiome and oral cavity cancer (OCC)

Emerging evidence from microbiome research has deepened our understanding of the microbial ecosystems implicated in OCC. Recent insights point to the oral microbiome as a potential contributor to both the initiation and progression of OCC through its influence on the local tumor microenvironment and host immune responses. This section explores the microbial associations with OCC, their potential mechanisms, and the impact of periodontal pathogens on disease pathogenesis. Microbial profiles and their role in the development and progression of OCC are summarized in Table 1.

**Table 1:** Microbial profiles and their role in the development and progression of oral cavity cancer. OCC: oral cavity cancer

Aspect	Key Microbial Profiles	Role in OCC
Microbial Associations	*Porphyromonas gingivalis*, *Fusobacterium nucleatum*, *Treponema denticola*	Promote tumorigenesis and cancer progression
	Pathogenic *Streptococcus* species (*Streptococcus anginosus*, *Peptostreptococcus*, S. genus)	Complex interplay between pathogenic and anticancer effects
Periodontal Pathogens	*Fusobacterium periodonticum*, *Parvimonas micra*, *Streptococcus constellatus*, *Haemophilus influenzae*	Contribute to malignant transformation
Sample-Specific Microbial Changes	Enriched in OCC: *Fusobacterium*, *Prevotella*, *Corynebacterium*	Reflect tissue-specific microbiome changes
	Depleted in OCC: *Streptococcus*, *Actinomyces*	
Early Microbial Shifts	*Fusobacterium*, *Leptotrichia*, and *Campylobacter* enriched in precancerous lesions	Potential for early detection and risk assessment

Microbial Associations with OCC

OCC is frequently associated with specific oral microorganisms, particularly periodontal pathogens. Research has identified several bacteria, such as *Porphyromonas gingivalis* (*P. gingivalis*), *Fusobacterium nucleatum*, and *Treponema denticola*, that exhibit a positive correlation with OCC. These microorganisms are often linked to the development and advancement of OCC, suggesting their potential role in tumorigenesis [12,13]. Interestingly, some members of the *Streptococcus* genus, typically associated with healthy oral flora, have been reported to show anticancer properties in vivo. However, more pathogenic *Streptococcus* species, including *Streptococcus anginosus* and *Peptostreptococcus*, exhibit a positive correlation with OCC, suggesting a more complex interaction between the microbiome and cancer [14]. This dual nature of *Streptococcus* bacteria further underscores the intricate connection between the oral microbiome and cancer development.

Periodontal Pathogens as Risk Factors for OCC

Periodontitis, a prolonged inflammatory disorder affecting the gums, is well-established as a major risk factor for the development of OCC. Several studies have indicated that bacteria linked to periodontal disease may be involved not only in the initiation of malignant changes but also in promoting the advancement of OCC [15,16]. The identification of specific pathogens, such as *Fusobacterium periodonticum*, *Parvimonas micra*, *Streptococcus constellatus*, *Haemophilus influenzae*, and *Filifactor alocis*, in OCC patients highlights the potential microbial signatures that could serve as early biomarkers for the disease [17]. Additionally, oral hygiene factors such as tooth loss and poor oral care, combined with the presence of bacteria commonly associated with periodontitis (e.g., *Prevotella tannerae*, *F. nucleatum*, *Prevotella intermedia*), have been identified as significant risk factors for OCC development [18,19]. The evidence points to microbial dysbiosis, especially in the setting of periodontal disease, as a potentially key driver in the early stages of OCC development.

The Impact of Sample Collection Methods on Microbial Diversity

Numerous factors, including the technique used for sample collection and the sample’s source, contribute to the diversity of the oral microbiome. Studies examining microbial profiles in OCC have emphasized the importance of considering tissue-specific variations in bacterial composition. For instance, research by Su et al. revealed that Fusobacterium species were significantly increased in OCC tumor tissue samples, while Streptococcus species showed a marked reduction [20]. Similarly, Sarkar et al. reported that tumor biopsies from OCC patients were enriched with specific bacteria, such as *Prevotella*, *Corynebacterium*, *Pseudomonas*, *Deinococcus*, and *Noviherbaspirillum* [21]. On the other hand, bacteria like *Actinomyces*, *Sutterella*, *Stenotrophomonas*, *Anoxybacillus*, and *Serratia* were found to be significantly less abundant in tumor tissues. These observations underscore the complex relationship between microbial populations and OCC. The variations in microbial profiles between healthy and diseased tissue samples may offer valuable insights into the progression of oral cancer and highlight the importance of considering tissue-specific microbiome dynamics.

Early Microbial Shifts in Precancerous Lesions

Microbial shifts within the oral cavity are evident both in OCC and during the early phases of tumorigenesis, particularly in premalignant conditions like oral leukoplakia. A study by Amer et al. investigated the microbiome in oral leukoplakia patients, a lesion with potential for malignant transformation [22]. They found a significant enrichment of *Fusobacterium*, *Leptotrichia*, and *Campylobacter* species in the leukoplakia lesions compared to healthy tissue. These microbial shifts suggest that alterations in the oral microbiome may precede cancer development and could be indicative of an early stage in the malignant transformation process. Additionally, Schmidt et al. reported an enrichment of *Bacteroidetes* and *Fusobacteria* in precancerous oral lesions, further supporting the idea that microbial alterations are not confined to invasive cancer but may begin earlier, during the precancerous phase [23]. These results suggest that characterizing the oral microbiome may serve as an informative biomarker for both early detection and risk evaluation in OCC.

Potential mechanisms for carcinogenesis

**Figure 2:**
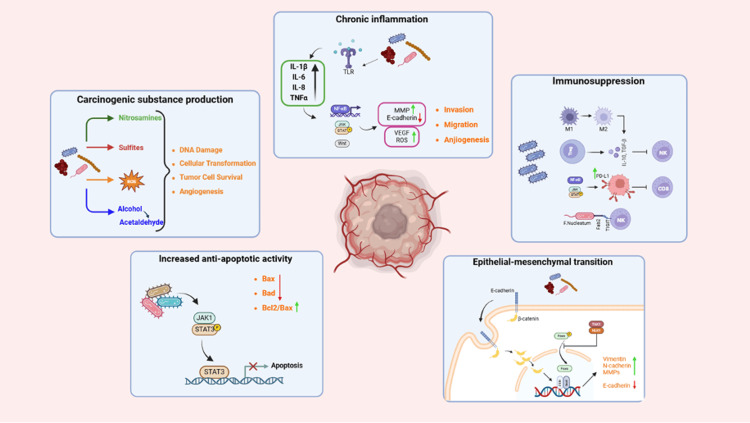
Microbiome-related mechanisms implicated in oral cavity carcinogenesis. This image was created with BioRender (https://biorender.com/).

The interplay between the microbiome and carcinogenesis has emerged as a critical area of research, particularly in the context of OCC. The microbial diversity within the oral environment has been shown to contribute to carcinogenic processes through several interrelated mechanisms. These include the production of carcinogenic substances, induction of chronic inflammation, suppression of immune responses, enhancement of anti-apoptotic activity, and promotion of epithelial-mesenchymal transition (EMT). Microbial metabolites such as nitrosamines, hydrogen sulfide, and acetaldehyde can directly induce genetic mutations and disrupt cellular homeostasis. Additionally, chronic inflammation triggered by bacterial components can activate oncogenic signaling pathways and facilitate tumor progression. The immune evasion strategies employed by certain pathogenic bacteria further exacerbate this process by impairing the immune system’s effectiveness in recognizing and eliminating malignant cells. Moreover, apoptosis resistance and EMT contribute to increased tumor invasiveness and metastatic potential. The subsequent sections delve into the underlying mechanisms and their contribution to the pathogenesis of OCC. Additionally, the potential mechanisms by which the oral microbiome may contribute to oral cavity carcinogenesis are outlined in Table 2. A graphical summary of these microbiome-associated carcinogenic pathways is presented in Figure 2.

**Table 2:** Summary of potential mechanisms for oral microbiome-associated carcinogenesis in oral cavity cancers.

Mechanism	Key Processes	Key Microbes
Carcinogenic Substance Production	Production of nitrosamines, hydrogen sulfide, and acetaldehyde (DNA damage, tumor survival).	*Streptococcus*, *Neisseria*, *Candida albicans*, *Fusobacterium nucleatum*, *Porphyromonas gingivalis*
Inflammation and Immune Suppression	Chronic inflammation (cytokines: IL-1β, IL-6, TNFα), immune evasion, and T-cell suppression.	*P. gingivalis*, *F. nucleatum*
Anti-Apoptotic Activity	Resistance to apoptosis via altered signaling pathways (e.g., JAK1-AKT-STAT3).	P. gingivalis
Epithelial-Mesenchymal Transition (EMT)	Promotion of mesenchymal markers, cell migration, and tumor invasiveness.	*F. nucleatum*, *P. gingivalis*, *Streptococcus gordonii*

Carcinogenic Substance Production

In parallel with gastrointestinal malignancies, the oral microbiota can synthesize carcinogenic metabolites such as nitrosamines, sulfites, oxides, and acetaldehyde, which are implicated in tumorigenesis [24]. These microbial metabolites are involved in key oncogenic processes, including cellular transformation, tumor survival, angiogenesis, and metastatic spread [25]. Nitrosamines, by binding to chemical bonds in DNA, can disrupt DNA replication and contribute to carcinogenesis. Strains of Candida albicans with high nitrosation potential have been isolated from precancerous lesions [26]. Additionally, *Firmicutes* and *Bacteroides* species, which are implicated in nitrosation processes associated with colorectal cancer, have also been identified within the oral microbiome [27]. Calenic et al. demonstrated that exposing gingival epithelial cells to hydrogen sulfide for 72 hours resulted in DNA fragmentation [28]. Notably, bacteria such as *P. gingivalis*, *P. intermedia*, and *F. nucleatum* are among those that produce hydrogen sulfide and induce DNA damage [29].

Another significant carcinogen produced by the microbiome is acetaldehyde (ACH). ACH is synthesized from alcohol by the enzyme alcohol dehydrogenase and is a metabolic byproduct of microorganisms. After alcohol consumption, ACH levels remain elevated in the oral cavity for up to three days [30]. In individuals who smoke, ACH levels are even higher. High concentrations of ACH synthesis are observed in bacterial cultures obtained from oral cancer and precancerous lesions [31]. Key bacteria involved in ACH production include *Streptococcus*, *Rothia*, *P. gingivalis*, and *Neisseria* species [32,33].

Inflammation and Immune Suppression

While the inflammatory response can play a protective role in carcinogenesis, increased inflammation is one of the key factors contributing to cancer development. The microbiome can trigger inflammatory mechanisms, and it would not be incorrect to assume that excessive inflammation induced by the microbiome leads to carcinogenesis [34]. Many bacteria in the oral cavity are known to induce chronic inflammation. Lipopolysaccharides, which are present in the cell walls of Gram-negative bacteria, as well as bacterial flagella and nucleic acids, are recognized by toll-like receptors (TLRs) on immune cells, resulting in an inflammatory response.

As inflammatory cells become activated, pro-inflammatory cytokines such as IL-1β, IL-6, and TNFα are released. In chronic periodontitis, the levels of these cytokines and matrix metalloproteinases (MMPs) are significantly elevated [34]. IL-1β stimulates endothelial cells to secrete vascular endothelial growth factor (VEGF), a key factor that promotes angiogenesis and facilitates tumor progression [35]. Additionally, IL-1β reduces E-cadherin expression and increases MMP secretion, promoting tumor invasion. During inflammation, MMPs increase both directly and via IL-1β, playing a crucial role in the invasion and migration of tumor cells by degrading the extracellular matrix and cell-cell junctions [36]. IL-6 and TNFα increase reactive oxygen species, which cause DNA damage [37], and TNFα also stimulates angiogenesis [38]. In addition to these effects, cytokines activate intracellular signaling pathways, including nuclear factor kB (NF-kB), Janus kinase (JAK)-signal transducer and activator of transcription (STAT), and WNT pathways [39]. Given the roles of these pathways in carcinogenesis, the role of microbiome-induced inflammation in cancer development can be better understood.

Another significant cytokine in *P. gingivalis*-mediated oral mucosa carcinogenesis is IL-8. The effects of IL8 in carcinogenesis are well known. It has been shown to induce angiogenesis and tumor cell proliferation, invasion, and chemotaxis, as well as to regulate apoptosis via STAT3 and NF-kB in various types of cancer. Ha et al. found that *P. gingivalis* increased the invasiveness of oral cancer cells by secreting IL8 and upregulating several MMPs [40]. *F. nucleatum* increases IL-6 and TNFα release via the TLR-MyD88 pathway [41] and activates the NLRP3 inflammasome, which leads to an increase in IL-1β release [42]. *P. gingivalis* enhances MMP-9 production and promotes cell invasion in OCC through the ERK1-Ets1 and NF-kB pathways [43].

Immune suppression also plays a crucial role in carcinogenesis. Many pathogens induce immune suppression. *P. gingivalis* increases PD-L1 expression in dendritic cells via the Akt-STAT3 pathway, leading to the suppression of cytotoxic T cells [44]. The upregulation of the immune checkpoint molecule B7-H4 by *P. gingivalis* is another mechanism that induces immune suppression. A study demonstrated that immune checkpoint inhibition suppressed *P. gingivalis*-induced carcinogenesis [45]. The gingipain molecule produced by *P. gingivalis* causes immune suppression by cleaving immunoglobulins and complement proteins [46]. Furthermore, *P. gingivalis* can reduce macrophage activity. Liu et al. co-cultured Cal-27 cells, *P. gingivalis*, and macrophages observed a decrease in the number of macrophages that phagocytosed Cal-27 cells in the presence of *P. gingivalis* after two hours [47].

*F. nucleatum* binds to the T cell immunoreceptor with immunoglobulin G (TIGIT) receptor via the Fap2 protein, inhibiting natural killer cells and facilitating the immune evasion of tumor cells [48]. In animal models, *F. nucleatum* has been shown to increase the number of M2 macrophages and myeloid-derived suppressor cells, which exert a significant suppressive effect on T cells [49].

Increased Anti-apoptotic Activity

The development of apoptosis resistance in tumor cells is one of the critical steps in carcinogenesis. The apoptosis resistance induced by *P. gingivalis* is a prime example of the role of the oral microbiome in this process. *P. gingivalis* alters intrinsic apoptotic activity through the JAK1-AKT-STAT3 signaling pathway [50,51]. Following *P. gingivalis* infection, an increase in BAD phosphorylation and the BCL2/BAX ratio is observed, leading to a reduction in apoptosis in epithelial cells [52]. Additionally, *P. gingivalis* modulates apoptosis by reducing P2X7 activation through the secretion of nucleoside diphosphate kinase.

Epithelial-Mesenchymal Transition (EMT)

EMT plays a crucial role in the development and progression of OCC. Bacteria such as *F. nucleatum* and *P. gingivalis* have been shown to promote the expression of mesenchymal markers while simultaneously reducing epithelial markers [53,54]. Additionally, these bacteria contribute to EMT induction, Snail activation, cell migration, and wound healing through the cytokines they produce [55]. *P. gingivalis* also upregulates the expression of the ZEB1 (zinc finger E-box binding homeobox 1) transcription factor, a key regulator of EMT. Elevated ZEB1 levels correlate with an increase in mesenchymal markers such as vimentin and MMP-9, as well as enhanced epithelial cell migration. Notably, *P. gingivalis* strains lacking the FimA fimbrial protein induce lower ZEB1 expression. In animal models, *P. gingivalis* infection has been shown to elevate ZEB1 levels in gingival tissues, and intracellular *P. gingivalis* has been detected in biopsy samples from OCC patients, suggesting that FimA-mediated ZEB1 expression may provide valuable insights into the role of *P. gingivalis* in OCC pathogenesis [56]. Furthermore, *P. gingivalis* can also enhance ZEB2 expression, which regulates EMT and inflammatory responses through pathways involving β-catenin and FOXO1 [57]. In contrast, *Streptococcus gordonii* suppresses ZEB1 and ZEB2 expression by activating the transforming growth factor β-activated kinase 1-Nemo-like kinase (TAK1-NLK) pathway, resulting in the inhibition of FOXO1 [57]. These findings suggest that periodontal pathogens can effectively induce EMT through various mechanisms.

## Conclusions

The interplay between microbial communities and cancer development has garnered considerable interest within both microbiology and oncology. Among various body sites, the oral cavity stands out due to its rich and diverse microbial ecosystem, which may have significant implications in cancer pathogenesis. However, sufficient evidence on this topic is still lacking, and further research is required. The etiology of OCC is complex and involves multiple factors. The most significant two risk factors, tobacco and alcohol, lead to carcinogenic changes caused by toxins and inflammation, which may also contribute to the mechanisms of oral cavity carcinogenesis through the microbiome.

As the mechanisms underlying microbial carcinogenesis become more clearly understood, potential therapeutic strategies targeting these pathways could be developed. Given that microbial factors play a role in both cancer initiation and treatment resistance, incorporating antimicrobial therapies into cancer treatment regimens should be considered. However, further clinical studies are needed to evaluate the feasibility of applying antimicrobial treatment in clinical practice. If the link between the microbiome and carcinogenesis is confirmed, microbial screening tests could be developed for early detection of certain types of cancer, particularly OCC. This could significantly improve prognosis through early diagnosis in OCC, which typically have poor survival rates in advanced stages. Additionally, antimicrobial treatments could potentially be integrated into anti-cancer therapies in the future, starting with OCC and extending to other cancer types.
